# Lenalidomide‐Associated Rhabdomyolysis Progressing to Bilateral Compartment Syndrome and Permanent Foot Drop

**DOI:** 10.1155/crom/6693120

**Published:** 2026-09-07

**Authors:** Karthik Iyer, Sanjna Chetan, Anoohya Arkala, Craig S. Siegel, Bhaskara Gadi, Cristian Popu, Vikram Oke, Briccio S. Cadiz

**Affiliations:** ^1^ Department of Internal Medicine, Mercy Hospital Jefferson, Festus, Missouri, USA; ^2^ Lake Erie College of Osteopathic Medicine, Erie, Pennsylvania, USA, lecom.edu

**Keywords:** adverse drug reaction, compartment syndrome, fasciotomy, foot drop, immunomodulatory agents, lenalidomide, multiple myeloma, rhabdomyolysis

## Abstract

Lenalidomide is widely used for the treatment and maintenance of multiple myeloma and is generally considered to have a favorable safety profile. However, severe skeletal muscle toxicity associated with lenalidomide has been rarely reported. We describe a 52‐year‐old man with multiple myeloma who developed acute bilateral lower‐extremity pain, cramping, weakness, and paresthesia while receiving lenalidomide therapy. Laboratory evaluation revealed marked rhabdomyolysis, and imaging and clinical findings were consistent with bilateral lower‐leg compartment syndrome. Despite emergent bilateral fasciotomies resulting in resolution of pain and normalization of creatine kinase levels, the patient developed persistent bilateral foot drop with residual sensory deficits. To our knowledge, this represents the first reported case of lenalidomide‐associated rhabdomyolysis progressing to bilateral compartment syndrome with permanent neurologic sequelae. This case underscores the importance of early recognition of muscle toxicity, prompt laboratory evaluation, and timely surgical intervention to mitigate irreversible functional impairment.

## Highlights

• Lenalidomide‐associated rhabdomyolysis is a rare but serious adverse effect.
• In this case, rhabdomyolysis progressed rapidly to bilateral compartment syndrome requiring emergent fasciotomy.
• Despite timely multidisciplinary intervention, permanent neurologic deficits occurred.

## 1. Introduction

Multiple myeloma (MM), the second most common hematologic cancer after non‐Hodgkin lymphoma, is a clonal plasma cell malignancy arising in the bone marrow [1]. For eligible patients with newly diagnosed MM, standard treatment consists of induction chemotherapy followed by high‐dose chemotherapy and autologous hematopoietic stem cell transplantation (auto‐HSCT) [2].

Lenalidomide, a second‐generation immunomodulatory agent, is a cornerstone of MM therapy across multiple disease phases. First approved in 2006 in combination with dexamethasone for relapsed or refractory MM, its clinical use has expanded to encompass frontline therapy for transplant‐ineligible patients and post‐auto‐HSCT maintenance therapy [3]. Multiple randomized trials and meta‐analyses have demonstrated that lenalidomide maintenance therapy significantly improves progression‐free survival following auto‐HSCT, establishing it as standard of care in this setting [4].

Overall, lenalidomide is considered well tolerated. Common adverse effects include venous thromboembolism, rash, fatigue, infections, and hematologic toxicities such as neutropenia and thrombocytopenia. Less frequent but serious adverse events, including second primary malignancies and cardiopulmonary complications, such as pulmonary embolism and deep vein thrombosis (DVT), have been reported [5]. While peripheral neuropathy is less common with lenalidomide compared with earlier immunomodulatory agents, severe skeletal muscle toxicity has been rarely described.

Rhabdomyolysis is a potentially life‐threatening syndrome characterized by skeletal muscle breakdown with release of intracellular contents, including creatine kinase (CK) and myoglobin, into the circulation. Systemic complications include acute kidney injury, electrolyte abnormalities, and cardiac arrhythmias [6]. In severe cases, muscle edema within closed fascial compartments can result in increased intercompartmental pressure and compromised tissue perfusion, establishing a pathophysiologic link between rhabdomyolysis and acute compartment syndrome [7]. The diagnosis of rhabdomyolysis is typically based on compatible clinical features and a serum CK level exceeding five times the upper limit of normal (approximately > 1000 U/L) [6].

Here, we report a rare and severe case of lenalidomide‐associated rhabdomyolysis complicated by bilateral lower‐extremity compartment syndrome and permanent neurologic deficits, highlighting an uncommon but devastating toxicity of a widely used therapeutic agent.

## 2. Case Presentation

A 52‐year‐old man with a history of MM status post‐auto‐HSCT, hypertension, and chronic kidney disease (CKD) stage 3B presented with progressive bilateral lower‐extremity pain and cramping. Symptoms were localized primarily to the anterior compartments of both lower legs and feet, present at rest, and rated as 8/10 in severity, resulting in marked limitation in ambulation. The patient denied recent trauma, strenuous exercise, fever, rash, or infectious symptoms.

**Figure 1:**
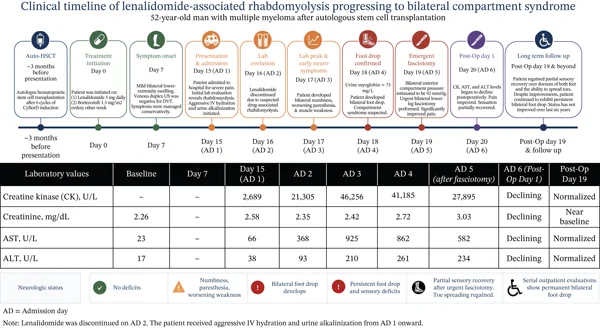
Timeline of case report. Clinical timeline illustrating the patient′s treatment course from auto‐HSCT through initiation of maintenance lenalidomide therapy, onset of symptoms and hospitalization, and long‐term neurologic outcome.

The patient′s oncologic history was notable for induction therapy with six cycles of cyclophosphamide and bortezomib, followed by auto‐HSCT 3 months prior to lenalidomide therapy. Post‐transplant, he was initiated on maintenance therapy with lenalidomide 5 mg orally once daily and bortezomib 1.3 mg/m^2^ administered subcutaneously every 2 weeks. Concomitant medications at the time of admission are summarized in Table 1. Refer to Figure 1 and Table 2 for a timeline of the case report.

**Table 1:** Patient medication list at time of admission.

**Maintenance therapy and concomitant medications**
● Lenalidomide 5 mg orally once daily ● Bortezomib 1.3 mg/m^2^ subcutaneously every other week
**Prophylactic and supportive medications**
● Acyclovir 200 mg orally twice daily ● Trimethoprim–sulfamethoxazole (DS 800–160 mg) one tablet orally three times weekly (Monday, Wednesday, and Friday) ● Aspirin 81 mg orally once daily ● Ergocalciferol (vitamin D₂) 50,000 IU orally once weekly ● Sodium bicarbonate 1300 mg orally twice daily ● Amlodipine–benazepril 10/20 mg orally daily ● Omega‐3 fatty acids one capsule orally once daily ● Multivitamin with minerals one tablet orally once daily

**Table 2:** Timeline of case report. Clinical timeline summarizing the patient′s treatment course.

Time	Clinical event
~3 months prior to lenalidomide	Pt underwent auto‐HSCT following six cycles of CyBorD induction therapy for multiple myeloma.
Start of lenalidomide	Initiated lenalidomide therapy 5 mg daily and bortezomib every 2 weeks.
7 days after lenalidomide	Developed mild bilateral lower‐extremity swelling and cramping. No evidence of DVT, so the patient was treated as an outpatient.
15 days after lenalidomide (Admission Day 1)	Presented to the emergency department with severe bilateral leg pain and inability to ambulate. CK level of 2689 U/L.
Admission Day 2	CK increased to 21,305 U/L despite treatment. Lenalidomide discontinued.
Admission Day 3	CK peaked at 46,256 U/L. AST increased to 925 U/L and ALT to 210 U/L. Developed bilateral lower‐extremity numbness, paresthesia, and worsening weakness.
Admission Day 4	Developed bilateral foot drop. Compartment syndrome suspected.
Admission Day 5	Orthopedic evaluation and urgent bilateral fasciotomy
Postoperative course (Day 19)	Pain markedly improved. Persistent bilateral foot drop and sensory deficits remained despite normalization of CK.

Seven days after initiation of lenalidomide, the patient developed mild bilateral lower‐extremity swelling and cramping. Duplex ultrasonography demonstrated no evidence of DVT, and Homan′s sign was negative. Symptoms were initially managed conservatively with compression stockings, leg elevation, and intermittent aspirin use.

Fifteen days after starting lenalidomide, the patient presented to the emergency department with debilitating bilateral leg pain and inability to ambulate. On admission (Day 1), vital signs were notable for blood pressure of 155/96 mmHg, temperature of 98°F, respiratory rate of 20 bpm, and oxygen saturation of 98% on room air. Initial laboratory evaluation revealed rhabdomyolysis, with a CK level of 2689 U/L. Additional laboratory findings and urinalysis results are summarized in Tables 3 and 4, respectively.

**Table 3:** Summary of lab values. The table shows pertinent serum and urine lab values obtained before and after initiation of lenalidomide therapy. “ND” indicates the test was not done.

Lab test	Reference range	Units	Patient value before lenalidomide	Patient value Admission Day 1	Patient value Admission Day 2	Patient value Admission Day 3	Patient value Admission Day 4	Patient value Admission Day 5 (after fasciotomy)
Aspartate aminotransferase (AST)	5–35	U/L	23	66	368	925	862	582
Alanine aminotransferase (ALT)	7–56	U/L	17	38	93	210	261	234
GFR	≥ 60	mL/min/1.73 m^2^	31	26	29	28	25	22
Albumin	3.5–4.8	U/L	4.5	4.8	4.7	4.3	4.2	3.7
Creatinine	0.5–1.4	mg/dL	2.26	2.58	2.35	2.42	2.72	3.03
Potassium	3.6–5.0	mEq/L	3.7	3.8	3.8	4.1	4.2	4.1
Sodium	137–145	mEq/L	143	135	134	134	138	133
Calcium	8.9–10.4	mg/dL	9.6	8.7	8.8	8.8	8.6	8.1
BUN	7–21	mg/dL	23	15	15	29	38	37
Glucose	65–110	mg/dL	107	172	129	132	117	119
Creatine kinase (CK)	39–308	U/L	ND	2689	21,305	46,256	41,185	27,895
Myoglobin (urine)	0–1	mg/L	ND	ND	ND	ND	75	ND

**Table 4:** Urinalysis values upon admission. The table shows pertinent urinalysis lab values obtained on the 2nd day of hospital admission.

Lab value	Reference range	Patient value
Color	Pale to dark yellow	Pale yellow
Clarity	Clear	Clear
Specific gravity	1.003–1.035	1.005
pH UA	5.0–8.0	8.0
Leukocyte esterase	Negative	Negative
Nitrite	Negative	Negative
Protein	Negative	2+
Glucose	Negative	1+
Ketones	Negative	Negative
Urobilinogen	< 2.0 mg/dL	Normal
Bilirubin	Negative	Negative
Blood	Negative	3+
WBC	0–2/hfp	0–2/hfp
RBC	0–2/hfp	3–5/hfp
Bacteria	Negative/hfp	1+/hfp
Epithelial cells	0–2/hfp	0–5/hfp
Hyaline casts	0–2/hfp	0–2/hfp

The patient was initiated on aggressive intravenous hydration with 0.45% normal saline containing 100 mEq sodium bicarbonate at 150 mL/h for renal protection and urine alkalinization. On Admission Day 2, CK levels rose sharply to 21,305 U/L despite therapy. The patient reported minimal pain (1/10) and denied fever, chills, or urinary symptoms. Given the temporal association with drug initiation, lenalidomide‐associated rhabdomyolysis was suspected, and lenalidomide was discontinued. After careful review, none of the patient′s maintenance, prophylactic, or supportive medications have been reported to directly cause rhabdomyolysis leading to compartment syndrome.

**Figure 2:**
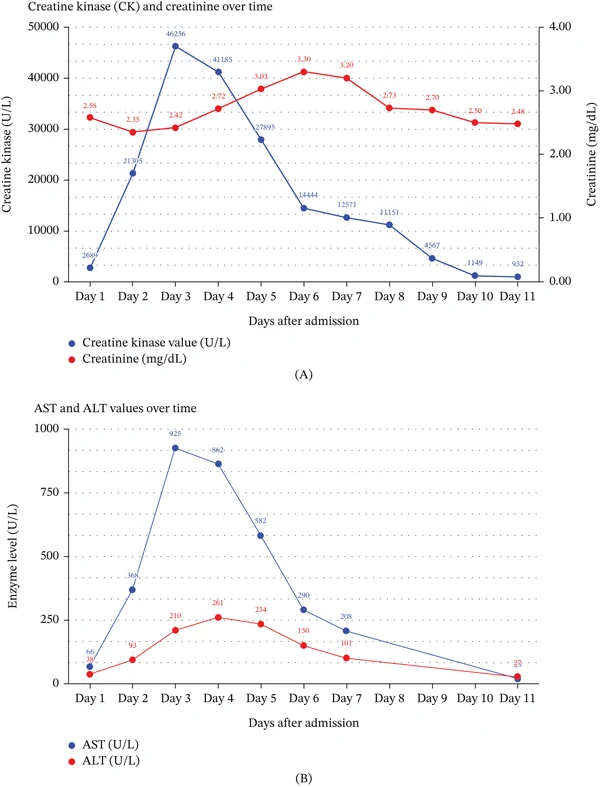
Serial changes in serum creatine kinase, creatinine, AST, and ALT levels since admission. (A) Creatine kinase (CK) reached a peak of 46,256 U/L on Day 3, consistent with severe rhabdomyolysis secondary to lenalidomide treatment. Creatinine levels peaked at 3.30 on Day 6. Prior to lenalidomide treatment, the patient′s baseline creatinine level ranged from 2.2 to 2.5. (B) This graph shows the elevated values of the patient′s liver enzymes. Peak levels were seen on Day 3 and Day 4 for AST and ALT, respectively. Fasciotomy occurred on Day 5. The patient′s liver enzymes gradually returned to baseline as the patient improved. AST, aspartate aminotransferase; ALT, alanine aminotransferase; U/L, units per liter.

On Admission Day 3, CK peaked at 46,256 U/L, accompanied by significant transaminase elevations (AST 925 U/L and ALT 210 U/L), as seen in Figure 2. The patient reported new‐onset bilateral lower‐extremity numbness and paresthesia, along with worsening weakness. On Admission Day 4, neurologic examination demonstrated bilateral foot drop, raising concern for evolving compartment syndrome. Urine myoglobin was 75 mg/L.

Orthopedic evaluation on Admission Day 5 revealed noncompressible anterior compartments bilaterally, diminished sensation over the anterior and lateral lower legs and dorsum of both feet, and absence of active ankle dorsiflexion. Posterior compartments remained soft and compressible. Compartment pressure measurements of the bilateral anterior compartments were estimated to be 92 mmHg (normal < 10 mmHg), confirming acute compartment syndrome.

Given the emergent nature of the condition, the patient underwent urgent bilateral lower‐leg fasciotomies with compartment decompression. Postoperatively, CK and liver enzyme levels progressively normalized, and pain significantly improved. However, motor and sensory deficits persisted, including bilateral foot drop, impaired dorsiflexion, and sensory loss over the dorsum of the feet.

At orthopedic follow‐up 19 days postoperatively, the patient demonstrated partial sensory recovery over the dorsal aspects of both feet and regained the ability to spread his toes. Despite these improvements, the patient continued to exhibit persistent bilateral foot drop and residual sensory deficits. During longitudinal follow‐up over the subsequent 6 years, physical examinations performed by the patient′s primary care physician consistently demonstrated persistent bilateral foot drop. These findings suggest long‐term neurologic sequelae related to the episode of rhabdomyolysis and compartment syndrome.

## 3. Discussion

Rhabdomyolysis is a clinical syndrome resulting from acute skeletal muscle injury characterized by sarcolemma disruption, intracellular calcium influx, and myocyte necrosis, leading to the release of CK, myoglobin, and electrolytes into the systemic circulation [8]. Although trauma, strenuous exertion, and ischemia are the most common precipitants, drug‐associated rhabdomyolysis is an atypical complication in oncology patients with altered metabolic reserve or underlying renal dysfunction [8].

Proposed mechanisms of lenalidomide‐mediated rhabdomyolysis include mitochondrial dysfunction, immune‐mediated myocyte injury, and dysregulation of intracellular calcium homeostasis, resulting in nontraumatic muscle damage [9, 10]. These effects may be amplified in post‐transplant patients due to recent exposure to high‐dose chemotherapy, baseline CKD, or impaired drug clearance. Severe skeletal muscle injury can lead to extensive edema and necrosis within closed fascial compartments, establishing a pathophysiologic link between rhabdomyolysis and the development of acute compartment syndrome, which is commonly caused by statins and trauma [6].

Management of rhabdomyolysis requires prompt recognition, aggressive intravenous hydration, and correction of electrolyte abnormalities to mitigate the risk of acute kidney injury [6]. When compartment syndrome is suspected, urgent surgical decompression via fasciotomy is essential to restore perfusion and prevent irreversible neuromuscular damage.

In this case, the patient developed rapidly progressive rhabdomyolysis with CK levels exceeding 46,000 U/L and markedly elevated anterior compartment pressures of 92 mmHg, necessitating emergent bilateral lower‐extremity fasciotomies. To our knowledge, this represents the first reported case of lenalidomide‐associated rhabdomyolysis complicated by bilateral compartment syndrome with permanent neurologic sequelae. Notably, most previously reported cases of lenalidomide‐associated rhabdomyolysis involved additional precipitating factors, including concomitant statin therapy, hypothyroidism, acute dehydration, or intercurrent infection.

A PubMed search using terms (“lenalidomide” AND “rhabdomyolysis”) OR (“lenalidomide” AND “compartment syndrome”) identified only a limited number of reported cases of rhabdomyolysis temporally associated with lenalidomide therapy, as seen in Table 5. In two separate reports published by Shahan et al. and Urata et al., rhabdomyolysis occurred in patients receiving concurrent statin therapy, limiting definitive attribution to lenalidomide alone [11, 12]. A case report published by Rathore and Singh described a patient in the setting of acute dehydration who developed rhabdomyolysis and subsequent acute kidney injury [13]. Blair et al. reported rhabdomyolysis following lenalidomide‐associated hypothyroidism [14]. A clinical trial published by Brown et al. compared the adverse effects of lenalidomide in a Phase 1 clinical trial. One out of nine patients developed rash and myalgias, initially thought to be due to influenza and concomitant use of simvastatin. Following recovery from influenza and discontinuing simvastatin, researchers rechallenged the patient with lenalidomide. The patient developed rhabdomyolysis, now confirmed by diagnostic studies, on Day 2 of treatment, providing stronger evidence for a causal relationship [15]. Importantly, none of these reported cases progressed to compartment syndrome or resulted in permanent neurologic deficits.

**Table 5:** List of comparable cases. All patients in this table developed confirmed rhabdomyolysis with a suspected etiology of lenalidomide therapy.

Studies	Patient demographics	Days on lenalidomide treatment	Other medications for multiple myeloma	CK value (U/L)	Serum creatinine (mg/dL)	Compartment syndrome	Other potential contributing factors
Shahan et al.	49 F	5 mg: 3 months 10 mg: 8 days^a^	Fludarabine and rituximab	1051	2.39	No	Pravastatin
Urata et al.	77 M	5 days	Dexamethasone	16,126	1.49	No	Pravastatin
Rathore and Singh	75 F	—	Dexamethasone and bortezomib	4489	—	No	Acute dehydration
Blair et al.	49 M	2 years	Dexamethasone and bortezomib	11,041	1.80	No	Hypothyroidism
Brown et al.	59 M	Round 1: 19 days Round 2: 2 days^b^	Fludarabine and rituximab	Grade 4 CK (> 10× ULN)	—	No	Influenza
Our patient	52 M	16 days	Dexamethasone and bortezomib	46,256	3.30	Yes	Stage 3B CKD, post‐auto‐HSCT status, and bortezomib

*Note:* “—” indicates no value given.

Abbreviation: ULN, upper limit of normal.

^a^The patient was treated with lenalidomide 5 mg for 3 months. The dose was increased to 10 mg 8 days prior to rhabdomyolysis.

^b^The patient was treated with lenalidomide for 19 days before developing adverse effects. The drug was discontinued. The patient was rechallenged and developed rhabdomyolysis on Day 2.

Collectively, these reports demonstrate that lenalidomide‐associated rhabdomyolysis, while rare, is a reproducible adverse event with considerable heterogeneity in timing, severity, and clinical outcomes. Onset has been reported from days to years following treatment initiation, suggesting an idiosyncratic rather than dose‐dependent mechanism. Although concomitant medications and metabolic stressors are frequently present, no consistent predisposing factor has been identified. Dexamethasone is the most commonly coadministered agent across reported cases, raising the possibility of synergistic toxicity, although causality remains unproven.

Although the temporal relationship between lenalidomide initiation and symptom onset suggests a possible association, concomitant factors, including bortezomib exposure, underlying stage 3B CKD, and the recent post‐auto‐HSCT state, may also have contributed by influencing susceptibility to muscle injury, drug pharmacokinetics, or physiologic reserve. Alternative etiologies of rhabdomyolysis were considered during the diagnostic evaluation. The patient had no clinical evidence of viral illness, recent trauma, strenuous exertion, or inflammatory myopathy, and there were no significant electrolyte abnormalities or metabolic disturbances to account for the severity of muscle injury. Thyroid dysfunction was considered clinically unlikely based on the patient′s presentation; however, thyroid function testing was not obtained, and therefore, this alternative etiology was not formally excluded. While lenalidomide remains the most likely precipitating factor based on the temporal sequence, the potential contribution of these additional clinical factors should be considered when interpreting the findings. Based on the WHO–UMC Causality System assessment criteria, the adverse event in our patient would be classified as a probable association [16].

Further, the elevated aminotransferase levels observed in this patient may have primarily reflected skeletal muscle injury associated with rhabdomyolysis rather than intrinsic hepatic injury. Marked elevations in CK accompanied by increased serum aminotransferases have been well described in rhabdomyolysis and may mimic hepatocellular injury [17]. In our patient, there was no documented history of liver disease. Prior laboratory evaluations had not demonstrated elevated transaminase levels, and no clinical features suggestive of primary hepatic injury were identified. The marked elevation in CK together with the clinical presentation of rhabdomyolysis therefore supports skeletal muscle injury as the most likely source of the transaminase elevation.

This case highlights the need for heightened clinical vigilance in patients receiving lenalidomide, particularly those with baseline renal dysfunction or recent exposure to high‐dose chemotherapy. Early recognition of neuromuscular symptoms, prompt laboratory evaluation, and a low threshold for orthopedic consultation are critical, as delayed intervention may result in irreversible functional impairment. Further investigation into potential drug interactions and mechanistic pathways may help identify patients at increased risk and guide preventive monitoring strategies.

## 4. Conclusion

Drug‐associated rhabdomyolysis, which may progress to acute compartment syndrome, is a rare but serious complication reported in association with lenalidomide. Markedly elevated CK levels, critically increased compartment pressures, and permanent bilateral foot drop illustrate the potential for irreversible functional impairment. Clinicians should remain vigilant for neuromuscular symptoms or rising CK and ensure early recognition and timely multidisciplinary management, including surgical intervention when indicated, to prevent lasting neurologic sequelae.

## 5. Clinical Relevance

Clinicians should maintain a high index of suspicion for skeletal muscle toxicity in patients treated with lenalidomide. Early CK monitoring and timely multidisciplinary evaluation may prevent progression to compartment syndrome and permanent neurologic deficits.

**Nomenclature**
AD: admission day
ALT: alanine aminotransferase
AST: aspartate aminotransferase
Auto‐HSCT: autologous hematopoietic stem cell transplantation
CK: creatine kinase
CKD: chronic kidney disease
CyBorD: cyclophosphamide, bortezomib, and dexamethasone
DVT: deep vein thrombosis
FDA: Food and Drug Administration
MM: multiple myeloma
U/L: units per liter

## Funding

No funding was received for this manuscript.

## Disclosure

The authors reviewed and edited the AI‐generated suggestions and take full responsibility for the final content.

## Consent

Written informed consent was obtained from the patient for the publication of the case report. A copy of the written consent is available for review upon request.

## Conflicts of Interest

The authors declare no conflicts of interest.

## Data Availability

The data that support the findings of this study are available upon request from the corresponding author. The data are not publicly available due to privacy or ethical restrictions.
